# Supplementary material for: Structural and functional characterization of NEMO cleavage by SARS-CoV-2 3CLpro
Source: Nat Commun. 2022 Sep 8;13:5285. doi: 10.1038/s41467-022-32922-9 (PMC9453703; doi:10.1038/s41467-022-32922-9)
Supplement: Supplementary file 1 — Supplementary Information [file 41467_2022_32922_MOESM1_ESM.pdf]

Supplementary Information

Structural and functional characterization of NEMO cleavage by SARS-CoV-2 3CLpro

Content:

- Supplementary Methods
- Supplementary Tables 1-10
- Supplementary Figures 1-4

Supplementary Methods

Molecular dynamics simulations and binding affinity predictions

Preparation of systems for simulations

Structural alignments were done using the MultiSeq[1] plugin within VMD[2]. Missing residues were added using coordinates of backbone atoms from the structure of SARS-CoV-2 3CLpro C145S variant. CHARMM-GUI[3] was used to model the missing side chains, to replace mutated residues by native residues, and to prepare simulation systems and inputs. [1] Structural water molecules were kept in the built systems.

The simulation boxes were generated by defining a solvation layer of 10 Å minimum thickness around the protein complex. 0.15 M KCL was used to establish electroneutrality. Protonation states for all systems were defined based on the neutron crystallographic structure of SARS-CoV-2 3CLpro (PDB id 7JUN) [4], which informs fine atomic details at a near-physiological pH (6.6).

Molecular dynamics simulations for classical analysis and for conformation selection

GROMACS-2020 [5] was used to run all simulations on the Summit supercomputer at the Oak Ridge Leadership Computing Facility. For all systems, initial energy minimization was performed with steepest descent for 5000 steps. The particle mesh Ewald method[6] was used for the treatment of periodic electrostatic interactions, using a cutoff distance of 12 Å. The Lennard–Jones potential was smoothed over the cutoff range of 10–12 Å. LINCS[7] was used to constrain all bonds involving hydrogen atoms. CHARMM36m[8–10] and TIP3P[11] force fields were used to describe protein and water molecules, respectively. Hydrogen mass repartitioning[12] was applied to the full 3CLpro-$NEMO_{227\text{-}234}$ systems to accelerate sampling as it allows using a 4 fs integration time step.

In the simulations for the MD/ML approach applied to the 3CLpro-$NEMO_{227-234}$ systems, velocity Langevin dynamics was performed using a friction constant of 1 ps^{-1}. An initial equilibration phase of about 5 ns for SARS-CoV-2 3CLpro and 24 ns for the other *betacoronaviruses* 3CLpro was conducted for a gradual increase in temperature up to 320.15 K and gradual release of position restraint harmonic potentials applied to $C_α$ atoms of 3CLpro and the NEMO-peptide. To allow the box size to adjust, most of the equilibration phase was conducted in the NpT ensemble, using the Berendsen barostat[13] applying a compressibility of 4.5 X 10^{-5} bar^{-1} and a time constant of 1.0 ps. In the last 1 ns of equilibration, the barostat was switched off and the next steps were carried out in the NVT ensemble. In the final step of equilibration, the atomic velocities were reinitialized independently from a Maxwell-Boltzmann distribution using random numbers of seed.

Following equilibration, five independent restrained MD simulations were performed for each system for conformational sampling and selection. Flat-bottom harmonic restraint potentials were applied to all $C_α$ atoms of 3CLpro, using a force constant of 0.25 kcal/mol/$Å^2$ and a flat-bottom width of 4 Å. Harmonic position restrain potential were kept at the $C_α$ atoms of Gln229, Leu230, and Gln231 in NEMO using a force constant of 0.05 kcal/mol/$Å^2$.

Visual Molecular Dynamics software suite (VMD, version 1.9.4a48)[14] was used for visual analysis. The Hbonds plugin in VMD was used to compute statistics of hydrogen bond interactions. The geometric criteria adopted are a cutoff of 3.0 Å for donor-acceptor distance and 20° for acceptor-donor-H angle. VMD was also used to compute distances and $C_α$ RMSD of selected atoms to a reference structure. Average number of contacts was computed using the mindist utility of GROMACS and a distance cutoff between $C_α$ atoms of 8 Å. The main contacts were identified using the Timeline plugin in VMD. Grace was used for the timeline plots (https://plasma-gate.weizmann.ac.il/Grace/).

To generate the illustrative representation of 3CLpro bound to the NEMO dimer (aa. 208-256; Figure 3b), a complex of 3CLpro with a long construct of human NEMO was built using Schrödinger Bioluminate Version 2019-4 [15]. The coordinates of the 7-residue fragment of NEMO (residues 227-233) from the

crystallographic structure of the porcine epidemic diarrhea virus (PEDV) 3CLpro bound to $NEMO_{227-233}$ (PDB id 5ZQG) and the coiled coil structure of NEMO from PDB ID 6MI3 [16] were used.

Binding affinity predictions

All FMO-DFTB/PCM calculations were carried out using the GAMESS v. 2020 program.[17] In the machine learning-based rescoring, the affinities/free energies corresponding to the seven features were obtained with the following freely available software: X-Score [18], DSX [19], RF-Score-VS [20], Deltavina [21], RF-Score-3 [22] , GalaxyDockBP2 [23], and Cyscore [24]. Autodocktools [25] was used to prepare the inputs for molecular re-docking.

Supplementary Tables

Supplementary Table 1. Comparison of distances between Thr26 and Thr190 in ligand-free and ligand-bound 3CLpro structures. Comparison of several 3CLpro structures indicate that the presence of hydrogen bonds (HB) formed between a substrate and both Thr190 and Thr26 is associated with a decrease in the distance (D) between these residues. C_α atoms were used to measure the distances. 7T2T, 7T2U, and 7T2V are the structures solved in this study. These structures were compared to both cryogenic- and room-temperature crystallography structures solved in complex with substrate peptides to assess what combination of hydrogen bonds formed between Thr26/Thr190 and the substrate peptide causes the decrease in distance between these two residues. We addressed this by selecting, for the cryogenic conditions, a peptide-bound C145A 3CLpro mutant (7N6N) and WT (7KPH) and C145S substrate-free (7N5Z) 3CLpro structures. For room temperature conditions, a WT 3CLpro structure (7JUN) and a C145A 3CLpro structure bound to an N-terminal peptide (7N89) were used. There is no available structure of substrate-free C145A 3CLpro solved at room-temperature for comparison. Room-temperature inhibitor-bound 3CLpro structures (telaprevir/boceprevir-bound WT 3CLpro) were chosen to demonstrate that binding in the active site groove without H-bonding to both Thr26 and Thr190 does not cause the decrease in distance observed in structures where these interactions are formed. In the table, '╳' indicates that there is no hydrogen bond is formed between the substrate and Thr26 or Thr190 and '✓' indicates that there is a hydrogen bond between the substrate and Thr26 or Thr190.

Temperature	3CLpro, ligand	PDB_ID	D, T26-T190 (Å)	HB with T26	HB with T190
Cryo	WT	7T2T	21.7	×	×
	C145S	7T2V	21.3, 21.5	×	×
	C145S, C-terminal tail	7T2V	21.1, 21.7	×	✓
	C145S, hNEMO$_{226\text{-}235}$	7T2U	20.3, 20.5	✓	✓
	C145S-hNEMO, C-terminal tail	7T2U	21.2, 21.2	×	×
Room	C145A, N-terminal peptide	7N89[26]	20.8, 20.9	✓	✓
	WT	7JUN[4].	21.0	×	×
	WT, Telaprevir	7LB7[27]	22.0	×	×
	WT, Boceprevir	6XQU[28]	21.7	×	×
Cryo	WT	7KPH[29]	21.3	×	×
	C145S	7N5Z[29]	21	×	×
	C145A, N-terminal peptide	7N6N[29]	21.7	×	✓
	C145A, C-terminal tail	7N6N[29]	21.6	×	×

Supplementary Table 2. Hydrogen bond interactions computed from molecular dynamics simulations of the SARS-CoV-2 3CLpro-NEMO bound complex. The average of occurrence (AVG) and its standard deviation (SD) are shown as the percentage of simulation time steps in n=5 independent MD runs of 100 ns each. Corresponding pairs of residues as well as atom name are identified ([residue]_[atom]_[side or main chain]).

**3CLpro**	**NEMO**	**AVG**	**SD**
Glu166_N/O_main	Gln229_O/N_main	93	2
Thr26_N/O_main	Ala233_O/N_main	88	4
Thr190_O_main	Ala228_N_main	63	15
Gly143_N_main	Gln231_O_main	41	8
His164_O_main	Gln231_N_main	36	20
Gln189_N_side	Ala228_O_main	30	8
His163_Nε2_side	Gln231_O_side	33	16
Cys145_N_main	Gln231_O_main	30	3
Asn142_N_side	Val232_O_main	21	4

Supplementary Table 3. Ranking of predicted hNEMO$_{227\text{-}234}$-binding affinities to 3CLpro from *betacoronaviruses* using quantum mechanics (QM)- and molecular dynamics/machine learning (MD/ML)-based methods. The MD/ML predicted binding affinities using molecular dynamics conformers are presented for five machine learning methods, namely support-vector machine (SVM), gradient-boosted trees (BT; scaled and unscaled*), and random forest (RF; scaled and unscaled*). The values of the predicted affinities are unitless, as they are expressed as -log(K_d). Quantum mechanics (QM) values of internal binding energy were computed employing the linear-scaling fragment molecular orbital density-functional tight-binding (FMO-DFTB) and are shown in kcal/mol. *Unscaled refers to the fact that unscaled, or unnormalized, features were used in the training.

3CLpro	MD/ML					QM
				Unscaled		
	SVM	BT	RF	BT	RF	FMO-DFTB
SARS2	8.28	8.24	8.48	6.71	6.42	-71.0
HKU1	7.18	6.99	7.06	6.67	6.28	-53.8
SARS1	6.71	6.18	6.18	6.58	6.23	-60.2
MERS	5.48	4.81	4.81	6.44	6.08	-50.9

Supplementary Table 4. Ranking of machine learning(ML)-predicted ranking of $hNEMO_{227\text{-}234}$-binding affinities to 3CLpro from *betacoronaviruses* using energy minimized-only structures. The ML-predicted rankings of binding affinities using energy minimized structures are presented for five methods, namely support-vector machine (SVM), gradient-boosted trees (BT; scaled and unscaled*), and random forest (RF; scaled and unscaled). Unscaled refers to the fact that unscaled, or unnormalized, features were used in the training.

Ranking	ML Model				
				Unscaled	
	SVM	BT	RF	BT	RF
1°	SARS1	SARS1	SARS1	SARS1	HKU1
2°	HKU1	SARS2	SARS2	SARS2	SARS1
3°	SARS2	MERS	HKU1	HKU1	MERS
4°	MERS	HKU1	MERS	MERS	SARS2

Supplementary Table 5. Comparison of interactions in the substrate-binding site of the hNEMO peptide-bound 3CLpro C145S with other peptide-bound 3CLpro structures. Changes in hydrogen bond (HB) interactions or hydrophobic contacts (HC) in each subsite of 3CLpro for different substrates are shown. The symbol "=" means that there is no difference in interaction profile relative to the reference structure (hNEMO peptide-bound 3CLpro C145S). Free water molecules are referred to as "W," while 3CLpro-bound water molecules are referred to as "W*". The strikethrough of residues correspond to those that interact via HB or HC with the substrate in the reference structure but do not make the same interaction in the compared structure. Excluded subsites are indicated with "X". The structures used for this comparison are as follows: 7JOY, SARS-CoV-2 3CLpro C145A in complex with its C-terminal residues[30]; 7N89, SARS-CoV-2 3CLpro C145A in complex with an N-terminal peptide[26]; 2Q6G, SARS-CoV 3CLpro H41A in complex with an N-terminal peptide[31]; 7N6N, SARS-CoV-2 3CLpro C145S in complex with N and C-terminal residues[29]; 7T2U, SARS-CoV-2 3CLpro C145S in complex with a hNEMO peptide and its C-terminal residues.

		Ligand (PDB_ID)					
**Subsite / Substrate**	**Interaction Type**	**C-ter (7JOY)**	**N-ter (7N89)**	**N-ter (2Q6G)**	**N-ter (7N6N)**	**C-ter (7N6N)**	**C-ter (7T2U)**
**S6/P6**	HB	Q192		=		Q192	Q192
	HC	=		=		=	=
**S5/P5**	HB	W*, W*	W*, P252, G251	W*	=	=	=
	HC	=	=	=	=	=	=
**S4/P4**	HB	~~T190~~, Q189	=	=	=	X	Q189, ~~T190~~
	HC	L167, Q192	=	M165	=	X	M165
**S3/P3**	HB	W*	=	=	=	=	=
	HC	=	E166	=	=	=	=
**S2/P2**	HB	~~Q189~~, W*, W*	W*,	=	X	~~Q189~~	~~Q189~~, W
	HC	=	=	=	X	=	=
**S1/P1**	HB	H41, F140 W ~~S144~~, ~~H164~~, E166	W*, F140, E166, ~~S144~~	F140, ~~S144~~	E166, ~~S144~~		W, ~~H164~~, E166
	HC	=	=	=	=		=
**S1'/P1'**	HB		X	W*, W, N142			
	HC		X	~~L27~~			
**S2'/P2'**	HB		=	W			
	HC		=	=			

		Ligand (PDB_ID)					
**Subsite / Substrate**	**Interaction Type**	**C-ter (7JOY)**	**N-ter (7N89)**	**N-ter (2Q6G)**	**N-ter (7N6N)**	**C-ter (7N6N)**	**C-ter (7T2U)**
**S3'/P3'**	HB		W	W			
	HC		M49	=			

Supplementary Table 6. Comparisons of 3CLpro recognition site at Gln231 in NEMO (aa. 226-235) from multiple animal species. Red font indicates the P1 site (Gln231 in human NEMO, hNEMO), light green highlights the reference sequence, and light purple, species harboring a different motif.

Order	Organism	Cleavage site $NEMO_{226-235}$	Reference NCBI
Primates	**Human** *Homo sapiens*	KLAQLQVAYH	NP_001093327.1
	**Chimpanzee** *Pan troglodytes*	KLAQLQVAYH	XP_016800099.2
	**Rhesus monkey** *Macaca mulatta*	KLAQLQVAYH	XP_001095498.2
	**Marmoset** *Callithrix jacchus*	KLAQLQVAYH	XP_008988365.2
	**Capuchin** *Sapajus apella*	KLAQLQVAYH	XP_032129057.1
	**Crab-eating macaque** *Macaca fascicularis*	KLAQLQVAYH	XP_005595059.1
Rodents	**Norway rat** *Rattus norvegicus*	KLAQLQAAYH	NP_954534.1
	**Thirteen-lined ground squirrel** *Ictidomys tridecemlineatus*	KLAQLQVAYH	XP_040143177.1
	**Chinese hamster** *Cricetulus griseus*	KLAQLQAAYH	XP_016836062.1
	**Mouse** *Mus musculus*	KLAQLQAAYH	NP_001154895.1
Carnivores	**Domestic ferret** *Mustela putorius furo*	KLAQLQVAYH	XP_012905560.1
	**Stoat** *Mustela erminea*	KLAQLQVAYH	XP_032187502.1
	**Domestic cat** *Felis catus*	KLAQLQVAYH	XP_004001080.1
	**Tiger** *Panthera tigris*	KLAQLQVAYH	XP_042830262.1
	**Dog** *Canis lupus familiaris*	KLAQLQVAYH	XP_038307227.1
Even-toed ungulates	**Horse** *Equus caballus*	KLAQLQVAYH	NP_001271462.1
	**Wild boar** *Sus scrofa*	KLAQLQVAYH	NP_001106524.1
	**Dromedary** *Camelus dromedarius*	KLAQLQVAYH	XP_031302174.1
Rabbit	**Rabbit** *Oryctolagus cuniculus*	KLAQLQVAYH	NP_001164965.1

Supplementary Table 6 (cont.). Comparisons of 3CLpro recognition site at Gln231 in NEMO (aa. 226-235) from multiple animal species. Red font indicates the P1 site (Gln231 in human NEMO, hNEMO), light green highlights the reference sequence, and light purple, species harboring a different motif.

Order	Organism	Cleavage site $NEMO_{226-235}$	Reference NCBI
Bats	**Common vampire** *Desmodus rotundus*	KLAQLQVAYH	XP_024406864.1
	**David's myotis** *Myotis davidii*	KLAQLQAAYH	XP_006754835.1
	**Great roundleaf** *Hipposideros armiger*	KLAQLQVAYH	XP_019489218.1
	**Black flying fox** *Pteropus alecto*	KLAQLQVAYH	XP_006904856.1
	**Natal long-fingered** *Miniopterus natalensis*	KLAQLQVAYH	XP_016077428.1
	**Brandt's** *Myotis brandtii*	KLAQLQAAYH	XP_005886157.1
	**Large flying fox** *Pteropus vampyrus*	KLAQLQVAYH	XP_039722945.1
	**Egyptian rousette** *Rousettus aegyptiacus*	KLAQLQVAYH	XP_015979392.2
	**Greater horseshoe** *Rhinolophus ferrumequinum*	KLAQLQVAYH	XP_032959395.1
	**Pale spear-nosed** *Phyllostomus discolor*	KLAQLQVAYH	XP_028378091.1
	**Big brown** *Eptesicus fuscus*	KLAQLQAAYH	XP_027991937.1
Pholidota	**Pangolin** *Manis javanica*	KLAQLQVAYH	XP_017521897.1

Supplementary Table 7. Crystallographic parameters, data collection and refinement statistics.

	3CLpro-NEMO	3CLpro C145S	3CLpro WT
**Crystallographic parameters**			
Space group	P1	P1	C2
Unit-cell dimensions	63.23Å, 67.77Å, 77.82Å<br>77.6˚, 90.0˚,72.9˚	61.59Å, 67.51Å, 77.65Å<br>102.1˚, 89.3˚,106.4˚	114.17Å, 53.44Å, 44.87Å<br>90.0˚, 103.0˚, 90.0˚
**Data collection statistics**			
Resolution limits (outer shell) (Å)	38.2 – 2.50(2.60-2.50)	38.05-2.47 (2.53-2.47)	38.9-1.45 (1.49-1.45)
No: of observed reflections (outer shell)	247591 (28289)	249278 (11167)	535143 (22778)
No: of unique reflections (outer shell)	41121 (4598)	40724 (2892)	46003 (3362)
Completeness (outer shell)	98.4 (97.1)	96.8 (93.6)	98.4 (98.4)
CC1/2 (outer shell)	97.9 (70.2)	99.4 (65.6)	99.9 (68.1)
R_{sym}[a] (outer shell) (%)	22.3 (114.6)	19.6 (102.0)	7.5 (156.6)
Mean I/σ(I) (outer shell)	5.9 (2.7)	8.3 (1.7)	16.0 (1.8)
**Refinement statistics**			
Resolution limits (Å)	37.9-2.50	38.05-2.47	38.9-1.45
Number of reflections (%) (|F|>2σ|F|)	41078 (98.7)	38686 (96.7)	43702 (98.4)
Reflections used for R_{free}	2058	2037	2301
R_{factor}[b] (%)	23.2	19.6	15.5
R_{free} (%)	27.6	23.7	20.0
Model contents (average B(Å^2))			
Protein atoms	9398 (36.2)	9425 (50.8)	2403 (33.4)
Peptide	154 (37.0)	0	0
Water molecules	110 (29.5)	46 (34.8)	227 (39.6)
RMS deviations			
Bond length (Å)	0.003	0.005	0.008
Bond angle (°)	0.63	1.14	1.47
Ramachandran (favored (%)/outliers)	96/2	94/6	98/0

[a] $R_{sym} = \Sigma|I_{avg}-I_i|/\Sigma I_i$

[b] R factor = $\Sigma|F_p - F_{pcalc}|/\Sigma F_p$, where F_p and F_{pcalc} are the observed and calculated structure factors; R_{free} is calculated with 5% of the data.

Supplementary Table 8. Molecular dynamics-based protocol of sampling for conformation selection of 3CLpro-NEMO$_{227\text{-}234}$ for the MD/ML approach. In the equilibration phases, there is a gradual change in temperature, in time step, and in the position restraint potentials. Atom velocities are re-distributed using five different seed numbers to initiate equilibration_6. With that, five independent trajectories are generated for conformational sampling. Applied potentials are either simple harmonic restraints (H) or flat-bottom potentials (FB). In NEMO$_{227\text{-}234}$, position restraints were applied to C_α atoms of residues 229-231.

Simulation phases	Position Restraints				Time Step	Total Time	Temperature
	3CLpro - C_α	Type	NEMO - C_α	Type			
**Equilibration_1**	0.50	H	0.25	H	1 fs	125 ps	100.15 K
**Equilibration_2**	0.25	H	0.25	H	2 fs	500 ps	200.15 K
**Equilibration_3**	0.14	H	0.25	H	2 fs	500 ps	250.15 K
**Equilibration_4**	0.05	H	0.25	H	2 fs	2 ns	300.15 K
**Equilibration_5**	0.05	H	0.14	H	4 fs	8 ns	310.15 K
**Equilibration_6**	0.14	FB	0.10	H	4 fs	1 ns (x 5)	320.15 K
**Sampling**	0.25	FB	0.10	H	4 fs	224 ns (x 5)	320.15 K

Supplementary Table 9. Classical molecular dynamics simulations of 3CLpro-NEMO$_{227-234}$ of different species. In the equilibration phases, there is a gradual change in temperature, in time step, and in the position restraint potentials. Atom velocities are re-distributed using five different seed numbers to initiate equilibration_6. With that, five independent trajectories are generated for conformational sampling. Applied potentials are either simple harmonic restraints (H) or flat-bottom potentials (FB). In NEMO$_{227-234}$, position restraints were applied to C_α atoms of residues 229-231.

Simulation phases	Position Restraints				Time Step	Total Time	Temperature
	3CLpro - C_α	Type	NEMO - C_α	Type			
**Equilibration_1**	0.50	H	0.25	H	1 fs	125 ps	100.15 K
**Equilibration_2**	0.25	H	0.25	H	2 fs	500 ps	200.15 K
**Equilibration_3**	0.14	H	0.25	H	2 fs	500 ps	250.15 K
**Equilibration_4**	0.05	H	0.25	H	2 fs	2 ns	300.15 K
**Equilibration_5**	0.05	H	0.14	H	4 fs	8 ns	310.15 K
**Equilibration_6**	0.25	FB	0.25	H	4 fs	1 ns (x 5)	310.15 K
**Production***	-	-	-	-	4 fs	112 ns (x 5)	310.15 K

*The five production runs of SARS-CoV-2 bound to human or mouse NEMO227-234 were extended to 192 ns.

Supplementary Figures

```
sp|O88522|NEMO_MOUSE      MNKHPWKNQLSEMVQPSGGPAEDQDMLGEESSLGKPAMLHLPSEQGTPETLQRCLEENQE
sp|Q9Y6K9|NEMO_HUMAN      MNRHLWKSQLCEMVQPSGGPAADQDVLGEESPLGKPAMLHLPSEQGAPETLQRCLEENQE
                          **:* **.**.********** ***:*****.**************:*************

                                                 83
sp|O88522|NEMO_MOUSE      LRDAIRQSNQMLRERCEELLHFQVSQREEKEFLMCKFQEARKLVERLSLEKLDLRSQREQ
sp|Q9Y6K9|NEMO_HUMAN      LRDAIRQSNQILRERCEELLHFQASQREEKEFLMCKFQEARKLVERLGLEKLDLKRQKEQ
                          **********:************.***********************.******: *:**

sp|O88522|NEMO_MOUSE      ALKELEQLKKCQQQMAEDKASVKAQVTSLLGELQESQSRLEAATKDRQALEGRIRAVSEQ
sp|Q9Y6K9|NEMO_HUMAN      ALREVEHLKRCQQQMAEDKASVKAQVTSLLGELQESQSRLEAATKECQALEGRARAASEQ
                          **:*:*:**:***********************************: ****** **.***

                                                     205                       231
sp|O88522|NEMO_MOUSE      VRQLESEREVLQQQHSVQVDQLRMQNQSVEAALRMERQAASEEKRKLAQLQAAYHQLFQD
sp|Q9Y6K9|NEMO_HUMAN      ARQLESEREALQQQHSVQVDQLRMQGQSVEAALRMERQAASEEKRKLAQLQVAYHQLFQE
                          .********.***************.*************************.*******:

sp|O88522|NEMO_MOUSE      YDSHIKS-------SKGMQLEDLRQQLQQAEEALVAKQELIDKLKEEAEQHKIVMETVPV
sp|Q9Y6K9|NEMO_HUMAN      YDNHIKSSVVGSERKRGMQLEDLKQQLQQAEEALVAKQEVIDKLKEEAEQHKIVMETVPV
                          **.****       .:*******:***************:********************

                             304      313
sp|O88522|NEMO_MOUSE      LKAQADIYKADFQAERHAREKLVEKKEYLQEQLEQLQREFNKLKVGCHESARIEDMRKRH
sp|Q9Y6K9|NEMO_HUMAN      LKAQADIYKADFQAERQAREKLAEKKELLQEQLEQLQREYSKLKASCQESARIEDMRKRH
                          ****************:*****.**** ***********:.***..*:************

sp|O88522|NEMO_MOUSE      VETPQPPLLPAPAHHSFHLALSNQRRSPPEEPPDFCCPKCQYQAPDMDTLQIHVMECIE
sp|Q9Y6K9|NEMO_HUMAN      VEVSQAPLPPAPAYLSSPLALPSQRRSPPEEPPDFCCPKCQYQAPDMDTLQIHVMECIE
                          **..*.** ****: *  ***..************************************
```

Supplementary Figure 1. Predicted 3CLpro cleavage sites in mouse and human NEMO. Aligned sequences of mouse (*Mus musculus*) and human NEMO are shown. There is 87% identity between mouse and human NEMO. The five predicted 3CLpro recognition sites at Gln83, Gln205, Gln231, Gln304, and Gln313 in human NEMO are indicated with the red lines. In mouse NEMO, these corresponding sites are Gln83, Gln205, Gln231, Gln297, and Gln306.

a

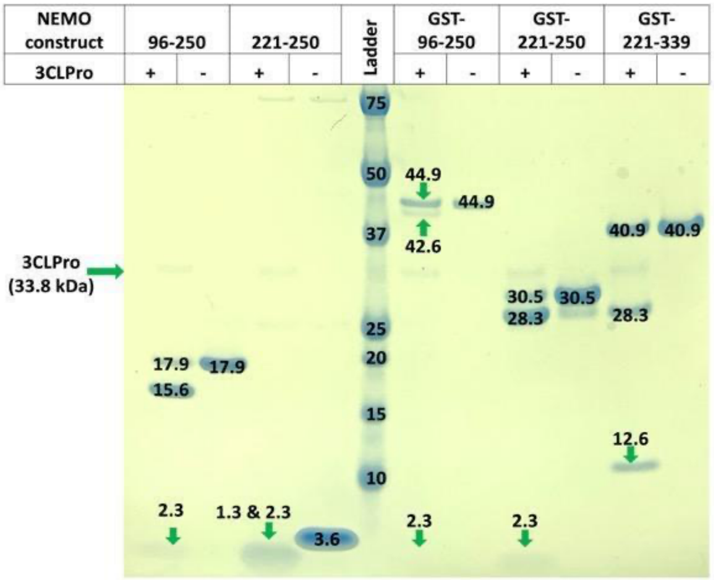

b

Lanes	96-250		221-250		GST-96-250		GST-221-250		GST-221-339	
3CLPro addition	(+)	(-)	(+)	(-)	(+)	(-)	(+)	(-)	(+)	(-)
Uncleaved (-)	17.87	17.87		3.59	44.85	44.85	30.54	30.5	40.85	40.85
Q205	12.70 + 5.19		N/A		39.68 + 5.19		N/A		N/A	
Q231	15.61 + 2.28		1.33 + 2.28		42.59 + 2.28		28.31 + 2.28		28.31 + 12.56	
Q304	N/A		N/A		N/A		N/A		36.75 + 4.25	
Q313	N/A		N/A		N/A		N/A		37.80 + 3.19	
Q205 & Q231	12.70 + 2.93 + 2.28		N/A		39.68 + 2.93 + 2.28		N/A		N/A	
Q231 & Q304	N/A		N/A		N/A		N/A		28.31 + 8.46 + 4.25	
Q304 + Q313	N/A		N/A		N/A		N/A		36.75 + 1.07 + 3.19	
Q231 + Q313	N/A		N/A		N/A		N/A		28.31 + 9.51 + 3.19	
Q231 + Q304 + Q313	N/A		N/A		N/A		N/A		28.31 + 8.46 + 1.07 + 3.19	

Supplementary Figure 2. 3CLpro cleaves mouse NEMO. a) SDS-PAGE following incubation of five truncations of mouse NEMO (0.1 mg/mL) with and without 3CLpro (0.25 μM). Proteolysis products are consistent with a single cleavage site following Gln231. **b)** Molecular weight (MW) estimates of NEMO and GST-NEMO fragments cleaved at Q205, Q231, Q304, and Q313 by SARS-COV2 3CLpro. MW values were calculated using EXPASY (https://web.expasy.org/compute_pi/). Fragments corresponding well with the SDS-PAGE gel patterns are highlighted in green and cleavage combinations not observed in yellow, indicating Q231 is the major cleavage site compared to Q205, Q304 and Q313. Q83 is not covered by the fragments tested in these experiments.

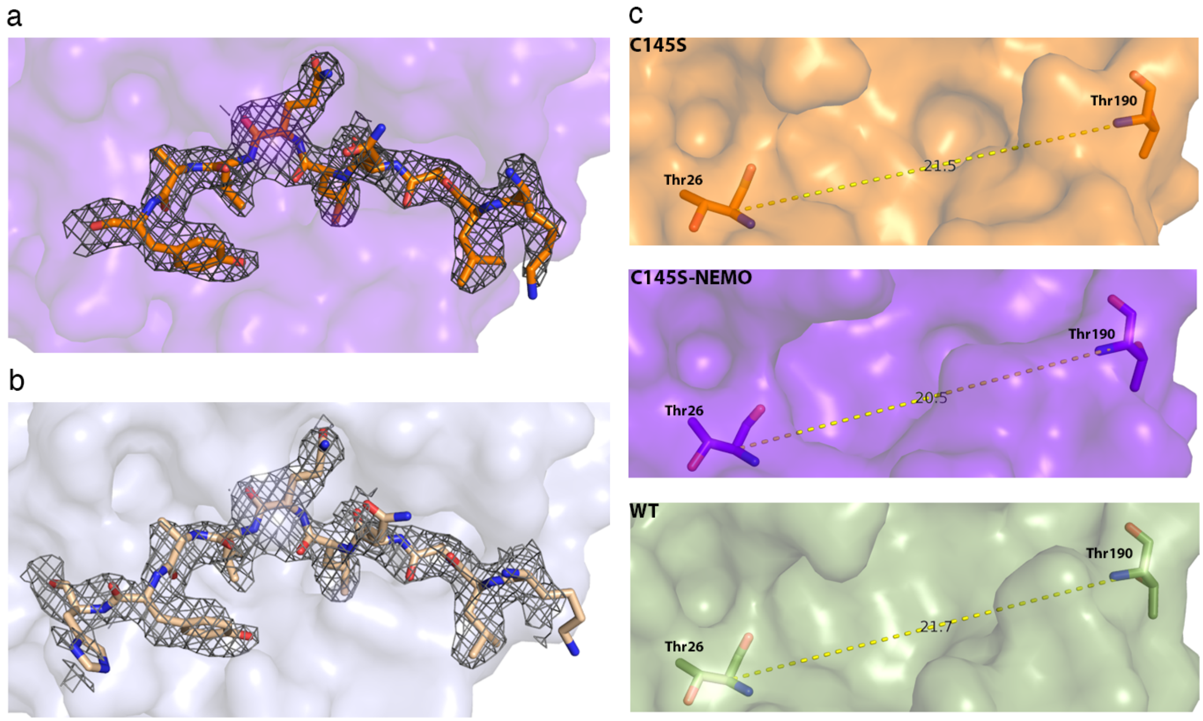

Supplementary Figure 3. Distance differences between Thr26 and Thr190 and NEMO peptide densities in 3CLpro. **a)** Electron density (composite OMIT map) around the $hNEMO_{226\text{-}234}$ peptide bound into the substrate-binding groove of chain B (purple surface) of 3CLpro C145S. The density is depicted as a gray mesh of width 0.5. The density also has a carve radius of 1.9 Å, and a contour level of 1.0 σ. His235 is not modeled in the hNEMO peptide bound into 3CLpro C145S chain B, because there is no density in the corresponding region. **b)** Electron density (composite OMIT map) around the $hNEMO_{226\text{-}235}$ peptide bound into the substrate-binding pocket of chain C (light blue surface) of 3CLpro C145S. The density is depicted as a gray mesh. The density also has a carve radius of 1.9 Å, and a contour level of 1.0 σ. The hNEMO peptide in **a** and **b** is depicted as sticks, where nitrogens are colored blue, oxygens are colored red and carbons are colored wheat or orange. **c)** The distances between the $C_α$ atoms of Thr190 and Thr26 (displayed as sticks) are displayed as labeled yellow dashed lines against surfaces of the 3CLpro structures solved in this study. Specifically, hNEMO-free SARS-CoV-2 3CLpro C145S (PDB_ID: 7T2V; orange surface and sticks of chain B), hNEMO-bound SARS-CoV-2 3CLpro C145S (PDB_ID: 7T2U; purple surface and sticks of chain B) and NEMO-free SARS-CoV-2 3CLpro

WT (PDB_ID: 7T2T; green surface and sticks). In the case of SARS-CoV-2 C145S with bound hNEMO, the hNEMO substrate is omitted for clarity and ease of comparison. In the representations of the hNEMO-free and hNEMO peptide-bound SARS-CoV-2 3CLpro C145S structures, only chain B is depicted. In SARS-CoV-2 3CLpro WT, there is only one chain in the asymmetric unit to depict.

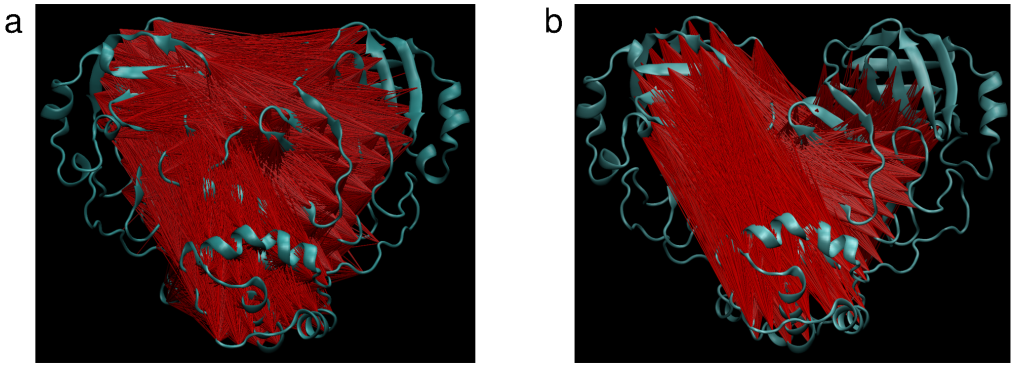

Supplementary Figure 4. Essential C_α cross-correlation analysis of SARS-CoV-2 3CLpro. Representative structural network of the 35707 pairs of residues that exhibit negative correlations (red sticks) with modulus greater than 0.85 identified in the MD trajectories of the **a)** SARS-CoV-2 3CLpro dimer (cyan) (PDB_ID 7JUN) and **b)** SARS-CoV 3CLpro dimer (PDB_ID 2DUC) [32]. We note that the asymmetry in the network of the latter structure likely reflects the fact that only one of its C-terminal tails is occupying the interfacial site.

Supplementary References

1. Roberts, E., Eargle, J., Wright, D. & Luthey-Schulten, Z. MultiSeq: unifying sequence and structure data for evolutionary analysis. *BMC Bioinformatics* **7**, 382 (2006).
2. Humphrey, W., Dalke, A. & Schulten, K. VMD: Visual molecular dynamics. *Journal of Molecular Graphics* vol. 14 33–38 (1996).
3. Jo, S., Kim, T., Iyer, V. G. & Im, W. CHARMM-GUI: a web-based graphical user interface for CHARMM. *J. Comput. Chem.* **29**, 1859–1865 (2008).
4. Kneller, D. W. *et al.* Unusual zwitterionic catalytic site of SARS-CoV-2 main protease revealed by neutron crystallography. *J. Biol. Chem.* **295**, 17365–17373 (2020).
5. Abraham, M. J. *et al.* GROMACS: High performance molecular simulations through multi-level parallelism from laptops to supercomputers. *SoftwareX* **1-2**, 19–25 (2015).
6. Darden, T., York, D. & Pedersen, L. Particle mesh Ewald: An N·log(N) method for Ewald sums in large systems. *J. Chem. Phys.* **98**, 10089–10092 (1993).
7. Hess, B., Bekker, H., Berendsen, H. J. C. & Fraaije, J. G. E. M. LINCS: A linear constraint solver for molecular simulations. *J. Comput. Chem.* **18**, 1463–1472 (1997).
8. Best, R. B. *et al.* Optimization of the additive CHARMM all-atom protein force field targeting improved sampling of the backbone φ, ψ and side-chain χ(1) and χ(2) dihedral angles. *J. Chem. Theory Comput.* **8**, 3257–3273 (2012).
9. MacKerell, A. D., Jr, Feig, M. & Brooks, C. L., 3rd. Improved treatment of the protein backbone in empirical force fields. *J. Am. Chem. Soc.* **126**, 698–699 (2004).
10. MacKerell, A. D. *et al.* All-atom empirical potential for molecular modeling and dynamics studies of proteins. *J. Phys. Chem. B* **102**, 3586–3616 (1998).
11. Jorgensen, W. L., Chandrasekhar, J., Madura, J. D., Impey, R. W. & Klein, M. L. Comparison of simple potential functions for simulating liquid water. *J. Chem. Phys.* **79**, 926–935 (1983).
12. Gao, Y. *et al.* CHARMM-GUI Supports Hydrogen Mass Repartitioning and Different Protonation

States of Phosphates in Lipopolysaccharides. *J. Chem. Inf. Model.* **61**, 831–839 (2021).

13. Berendsen, H. J. C., Postma, J. P. M., van Gunsteren, W. F., DiNola, A. & Haak, J. R. Molecular dynamics with coupling to an external bath. *J. Chem. Phys.* **81**, 3684–3690 (1984).
14. Humphrey, W., Dalke, A. & Schulten, K. VMD: visual molecular dynamics. *J. Mol. Graph.* **14**, 33–8, 27–8 (1996).
15. Zhu, K. *et al.* Antibody structure determination using a combination of homology modeling, energy-based refinement, and loop prediction. *Proteins* **82**, 1646–1655 (2014).
16. Barczewski, A. H., Ragusa, M. J., Mierke, D. F. & Pellegrini, M. The IKK-binding domain of NEMO is an irregular coiled coil with a dynamic binding interface. *Sci. Rep.* **9**, 2950 (2019).
17. Barca, G. M. J. *et al.* Recent developments in the general atomic and molecular electronic structure system. *J. Chem. Phys.* **152**, 154102 (2020).
18. Wang, R., Lai, L. & Wang, S. Further development and validation of empirical scoring functions for structure-based binding affinity prediction. *J. Comput. Aided Mol. Des.* **16**, 11–26 (2002).
19. Neudert, G. & Klebe, G. DSX: a knowledge-based scoring function for the assessment of protein-ligand complexes. *J. Chem. Inf. Model.* **51**, 2731–2745 (2011).
20. Wójcikowski, M., Ballester, P. J. & Siedlecki, P. Performance of machine-learning scoring functions in structure-based virtual screening. *Sci. Rep.* **7**, 46710 (2017).
21. Wang, C. & Zhang, Y. Improving scoring-docking-screening powers of protein-ligand scoring functions using random forest. *J. Comput. Chem.* **38**, 169–177 (2017).
22. Li, H., Leung, K.-S., Wong, M.-H. & Ballester, P. J. Improving AutoDock Vina Using Random Forest: The Growing Accuracy of Binding Affinity Prediction by the Effective Exploitation of Larger Data Sets. *Mol. Inform.* **34**, 115–126 (2015).
23. Baek, M., Shin, W.-H., Chung, H. W. & Seok, C. GalaxyDock BP2 score: a hybrid scoring function for accurate protein-ligand docking. *J. Comput. Aided Mol. Des.* **31**, 653–666 (2017).
24. Cao, Y. & Li, L. Improved protein-ligand binding affinity prediction by using a curvature-dependent surface-area model. *Bioinformatics* **30**, 1674–1680 (2014).

25. Morris, G. M. *et al.* AutoDock4 and AutoDockTools4: Automated docking with selective receptor flexibility. *J. Comput. Chem.* **30**, 2785–2791 (2009).

26. Kneller, D. W., Zhang, Q., Coates, L., Louis, J. M. & Kovalevsky, A. Michaelis-like complex of SARS-CoV-2 main protease visualized by room-temperature X-ray crystallography. *IUCrJ* **8**, 973–979 (2021).

27. Kneller, D. W. *et al.* Direct Observation of Protonation State Modulation in SARS-CoV-2 Main Protease upon Inhibitor Binding with Neutron Crystallography. *J. Med. Chem.* **64**, 4991–5000 (2021).

28. Kneller, D. W. *et al.* Malleability of the SARS-CoV-2 3CL Mpro Active-Site Cavity Facilitates Binding of Clinical Antivirals. *Structure* vol. 28 1313–1320.e3 (2020).

29. Noske, G. D. *et al.* A Crystallographic Snapshot of SARS-CoV-2 Main Protease Maturation Process. *J. Mol. Biol.* **433**, 167118 (2021).

30. Lee, J. *et al.* Crystallographic structure of wild-type SARS-CoV-2 main protease acyl-enzyme intermediate with physiological C-terminal autoprocessing site. *Nat. Commun.* **11**, 5877 (2020).

31. Xue, X. *et al.* Structures of two coronavirus main proteases: implications for substrate binding and antiviral drug design. *J. Virol.* **82**, 2515–2527 (2008).

32. Muramatsu, T. *et al.* SARS-CoV 3CL protease cleaves its C-terminal autoprocessing site by novel subsite cooperativity. *Proc. Natl. Acad. Sci. U. S. A.* **113**, 12997–13002 (2016).
