# Supplementary material for: Structural and functional characterization of NEMO cleavage by SARS-CoV-2 3CLpro
Source: Nat Commun. 2022 Sep 8;13:5285. doi: 10.1038/s41467-022-32922-9 (PMC9453703; doi:10.1038/s41467-022-32922-9)
Supplement: Supplementary file 3 — Description of Additional Supplementary Files [file 41467_2022_32922_MOESM3_ESM.pdf]

Supplementary Data 1

Description:

Input and output files of the AutoDock Vina calculations, energy minimized structures used as input in QM and ML calculations, DFTB-geometry optimized output structures, molecular dynamics snapshots selected using the MD/ML protocol, inputs to MD simulations, table of features used in the ML calculations, and unaveraged binding affinity predictions.
