# Supplementary material for: Structural and functional characterization of NEMO cleavage by SARS-CoV-2 3CLpro
Source: Nat Commun. 2022 Sep 8;13:5285. doi: 10.1038/s41467-022-32922-9 (PMC9453703; doi:10.1038/s41467-022-32922-9)
Supplement: Supplementary file 5 — Reporting Summary [file 41467_2022_32922_MOESM5_ESM.pdf]

nature portfolio

Corresponding author(s): Soichi Wakatsuki and Daniel Jacobson

Last updated by author(s): 08/11/2022

Reporting Summary

Nature Portfolio wishes to improve the reproducibility of the work that we publish. This form provides structure for consistency and transparency in reporting. For further information on Nature Portfolio policies, see our Editorial Policies and the Editorial Policy Checklist.

Statistics

For all statistical analyses, confirm that the following items are present in the figure legend, table legend, main text, or Methods section.

n/a	Confirmed	
☐	☒	The exact sample size (*n*) for each experimental group/condition, given as a discrete number and unit of measurement
☐	☒	A statement on whether measurements were taken from distinct samples or whether the same sample was measured repeatedly
☒	☐	The statistical test(s) used AND whether they are one- or two-sided<br>*Only common tests should be described solely by name; describe more complex techniques in the Methods section.*
☐	☒	A description of all covariates tested
☒	☐	A description of any assumptions or corrections, such as tests of normality and adjustment for multiple comparisons
☐	☒	A full description of the statistical parameters including central tendency (e.g. means) or other basic estimates (e.g. regression coefficient) AND variation (e.g. standard deviation) or associated estimates of uncertainty (e.g. confidence intervals)
☒	☐	For null hypothesis testing, the test statistic (e.g. *F*, *t*, *r*) with confidence intervals, effect sizes, degrees of freedom and *P* value noted<br>*Give P values as exact values whenever suitable.*
☒	☐	For Bayesian analysis, information on the choice of priors and Markov chain Monte Carlo settings
☒	☐	For hierarchical and complex designs, identification of the appropriate level for tests and full reporting of outcomes
☒	☐	Estimates of effect sizes (e.g. Cohen's *d*, Pearson's *r*), indicating how they were calculated

Our web collection on statistics for biologists contains articles on many of the points above.

Software and code

Policy information about availability of computer code

Data collection	BLU-ICE v5.1., BLEND version 0.6.23, GROMACS-2020. In Supplementary Information: Autodock-Vina and GAMESS v. 2022
Data analysis	MOLREP v. 11.7.03; 13.07.2020 and v. 11.9.02; 28.02.2022, Coot v. 0.9.6, REFMAC v. 5.8.0267 and v. 5.8.0349 , Phenix v. 1.20.1, GROMACS-2020. In Supplementary Information: VMD v. 1.9.4a48, X-Score v. 1.2.1, DSX, RF-Score-VS v. 1.0, Deltavina, RF-Score-3 v. 3.0, GalaxyDockBP2, and Cyscore v. 2.0

For manuscripts utilizing custom algorithms or software that are central to the research but not yet described in published literature, software must be made available to editors and reviewers. We strongly encourage code deposition in a community repository (e.g. GitHub). See the Nature Portfolio guidelines for submitting code & software for further information.

Data

Policy information about availability of data

All manuscripts must include a data availability statement. This statement should provide the following information, where applicable:

- Accession codes, unique identifiers, or web links for publicly available datasets
- A description of any restrictions on data availability
- For clinical datasets or third party data, please ensure that the statement adheres to our policy

Structural data that support the findings of this study have been deposited in RCSB PDB with PDB accession codes: 7T2T, 7T2U, 7T2V. The Source Data generated in the enzymatic assays is provided in Supplementary Data 1. Input and output files of the AutoDock Vina calculations, the energy minimized structures used as input in QM and ML calculations, the DFTB-geometry optimized output structures, the molecular dynamics snapshots selected using the MD/ML protocol, the inputs to molecular dynamics simulations, the table of features used in the ML calculations, and the unaveraged binding affinity predictions are provided in Supplementary Data 2. The raw MD trajectories are too large to be shared publicly, so we share inputs to reproduce our molecular dynamics simulations.

All the PDB datasets used in the study along with their accession-codes are in the manuscript.

Field-specific reporting

Please select the one below that is the best fit for your research. If you are not sure, read the appropriate sections before making your selection.

☒ Life sciences ☐ Behavioural & social sciences ☐ Ecological, evolutionary & environmental sciences

For a reference copy of the document with all sections, see nature.com/documents/nr-reporting-summary-flat.pdf

Life sciences study design

All studies must disclose on these points even when the disclosure is negative.

Sample size	For our MD/ML method, we performed restrained MD simulations of equilibrated 3CLpro-NEMO227-235 systems to sample 280,000 conformations. The protocol of MD simulations used for conformation selection is adaption of the MD-based refinement described in Heo et al. 2021 (https://doi.org/10.1021/acs.jctc.0c01238). Our sampling exceeds the original approach and, combined with machine learning it has been successfully used to predict relative binding affinities in benchmarking data. For classical MD simulations of 3CLpro-NEMO190-270 and 3CLpro-NEMO227-235, 5 x ~115 ns trajectories were generated. This simulation time was long enough to observe a remarkable deviation of NEMO190-270 from its original position due to the single substitution V232A. The simulation time is adequate to cover local conformational changes and indicate key interacting sites in 3CLpro-NEMO227-235 (https://doi.org/10.1016/j.coph.2010.09.008).
Data exclusions	No data was excluded from analysis.
Replication	NEMO cleavage assays were prepared in duplicate; Five independent restrained molecular dynamics (MD) simulations were performed for each 3CLpro-NEMO227-234 system. Five independent classical MD simulations were performed for each 3CLpro-NEMO190-270 system. We collected 8 data sets for the WT Mpro native structure. Six data sets were collected for the C145S mutant and 4 data sets for the Mpro-Nemo structure. 7 data sets of the WT Mpro were successful, as were all data sets for C145S (6 data sets) and Mprro-Nemo (4 data sets).
Randomization	Covariates were controlled using controlled data-collection conditions in both assays, crystallography and computational simulations. For crystallography data, we used CC1/2 values to assess data quality and highest resolution, using randomly selected half of reflections for consistency test (Karplus and Diederichs (2012) Science 336, 1030).
Blinding	Crystallographic Rfree factors are calculated by blinding 5% of diffraction data to assess the potential structure model bias (Kleywegt and Jones, Methods in Enzymology 277, 208-230 (1997)). Blinding is not relevant to the other part of the study, because we are making biochemical measurements of a particular enzyme and substrate of interest.

Reporting for specific materials, systems and methods

We require information from authors about some types of materials, experimental systems and methods used in many studies. Here, indicate whether each material, system or method listed is relevant to your study. If you are not sure if a list item applies to your research, read the appropriate section before selecting a response.

Materials & experimental systems

n/a	Involved in the study
☒	☐ Antibodies
☒	☐ Eukaryotic cell lines
☒	☐ Palaeontology and archaeology
☒	☐ Animals and other organisms
☒	☐ Human research participants
☒	☐ Clinical data
☒	☐ Dual use research of concern

Methods

n/a	Involved in the study
☒	☐ ChIP-seq
☒	☐ Flow cytometry
☒	☐ MRI-based neuroimaging
